# MST4 negatively regulates the EMT, invasion and metastasis of HCC cells by inactivating PI3K/AKT/Snail1 axis

**DOI:** 10.7150/jca.60008

**Published:** 2021-05-27

**Authors:** Mei-Juan Dian, Jing Li, Xiao-Ling Zhang, Zi-Jian Li, Ying Zhou, Wei Zhou, Qiu-Ling Zhong, Wen-Qian Pang, Xiao-Lin Lin, Tao Liu, Yi-An Liu, Yong-Long Li, Liu-Xin Han, Wen-Tao Zhao, Jun-Shuang Jia, Sheng-Jun Xiao, Dong Xiao, Jia-Wei Xia, Wei-Chao Hao

**Affiliations:** 1 Cancer Research Institute, School of Basic Medical Sciences, Southern Medical University, Guangzhou 510515, China.; 2 Institute of Comparative Medicine & Laboratory Animal Center, Southern Medical University, Guangzhou 510515, China.; 3 Radiotherapy Center, the First People's Hospital of Chenzhou, Xiangnan University, Chenzhou 423000, China.; 4 Department of Physiology, Faculty of Basic Medical Sciences, Guilin Medical University, Guilin 541004, China.; 5 State Key Laboratory of Organ Failure Research, Key Laboratory of Mental Health of the Ministry of Education, Guangdong-Hong Kong-Macao Greater Bay Area Center for Brain Science and Brain-Inspired Intelligence, Guangdong Province Key Laboratory of Psychiatric Disorders, Collaborative Innovation Center for Brain Science, Department of Neurobiology, School of Basic Medical Sciences, Southern Medical University, Guangzhou 510515, China.; 6 The third people's hospital of Kunming (The Sixth Affiliated Hospital of Dali University), Kunming 650041, China.; 7 Department of Gastrointestinal Oncology, The Third Affiliated Hospital of Kunming Medical University (Yunnan Cancer Hospital, Yunnan Cancer Center), Kunming 650118, China.; 8 Department of Pathology, the Second Affiliated Hospital, Guilin Medical University, Guilin 541199, China.; 9 Department of Oncology, The First Affiliation Hospital of Guangdong Pharmaceutical University, Guangzhou 510062, China.

**Keywords:** MST4, Hepatocellular carcinoma, Metastasis, Prognosis, EMT, PI3K/AKT, Snail1

## Abstract

**Background:** Hepatocellular carcinoma (HCC) is one of the most common cancers worldwide and has a poor prognosis due to the high incidence of invasion and metastasis-related progression. However, the underlying mechanism remains elusive, and valuable biomarkers for predicting invasion, metastasis, and poor prognosis of HCC patients are still lacking.

**Methods**: Immunohistochemistry (IHC) was performed on HCC tissues (n = 325), and the correlations between MST4 expression of the clinical HCC tissues, the clinicopathologic features, and survival were further evaluated. The effects of MST4 on HCC cell migratory and invasive properties *in vitro* were evaluated by Transwell and Boyden assays. The intrahepatic metastasis mouse model was established to evaluate the HCC metastasis *in vivo*. The PI3K inhibitor, LY294002, and a specific siRNA against Snail1 were used to investigate the roles of PI3K/AKT pathway and Snail1 in MST4-regulated EMT, migration, and invasion of HCC cells, respectively.

**Results:** In this study, by comprehensively analyzing our clinical data, we discovered that low MST4 expression is highly associated with the advanced progression of HCC and serves as a prognostic biomarker for HCC patients of clinical-stage III-IV. Functional studies indicate that MST4 inactivation induces epithelial-to-mesenchymal transition (EMT) of HCC cells, promotes their migratory and invasive potential *in vitro*, and facilitates their intrahepatic metastasis *in vivo*, whereas MST4 overexpression exhibits the opposite phenotypes. Mechanistically, MST4 inactivation elevates the expression and nuclear translocation of Snail1, a key EMT transcription factor (EMT-TF), through the PI3K/AKT signaling pathway, thus inducing the EMT phenotype of HCC cells, and enhancing their invasive and metastatic potential. Moreover, a negative correlation between MST4 and p-AKT, Snail1, and Ki67 and a positive correlation between MST4 and E-cadherin were determined in clinical HCC samples.

**Conclusions:** Our findings indicate that MST4 suppresses EMT, invasion, and metastasis of HCC cells by modulating the PI3K/AKT/Snail1 axis, suggesting that MST4 may be a potential prognostic biomarker for aggressive and metastatic HCC.

## Introduction

Hepatocellular carcinoma (HCC) is ranked as the sixth most common cancer and the fourth leading cause of cancer death 1. Due to the metastasis-related high intrahepatic recurrence rate, the long-term survival of patients with HCC is very poor 2. However, no effective treatment options to reduce the risk of metastasis-associated intrahepatic recurrence are available 3. Surgical resection and ablation are the frontline treatment choices for patients with early HCC 4, 5. Unfortunately, 70% of the cases would have tumor recurrence within the remaining liver at 5 years 6. Liver transplantation is an effective treatment to prevent intrahepatic recurrence, but the source of liver donors is limited 1, 3, 6. Sorafenib, a targeted therapeutic drug, is recommended for patients with advanced or intermediate-stage HCC 7. It is a multi-kinase inhibitor that blocks Raf signaling, VEGF, PDGF, and c-Kit, and has anti-proliferative and anti-angiogenic effects 8-11. Although it has been shown to be beneficial for HCC patients' survival 12, 13, it has also been proven to not prevent HCC tumor intrahepatic recurrence 14. Therefore, it is urgent to understand the cellular and molecular mechanism of HCC metastasis and to find potential molecules that can be used to predict the aggressive invasion, metastasis, and poor prognosis of HCC, so as to provide opportunities to prevent intrahepatic recurrence of this malignancy.

Metastasis is a complex multi-step process that involves the dissemination of cancer cells from the primary tumor; intravasation into the circulatory system; and subsequent colonization in the distant tissue to form metastatic foci 15. Intrahepatic metastasis (IM) is the most common hematogenous metastasis of HCC, which is usually caused by tumor cell dispersal via the portal vein 16. Epithelial-mesenchymal transition (EMT) is a process of cell transdifferentiation in which cells lose their epithelial characteristics and acquire mesenchymal phenotype 17, 18. For HCC progression, EMT is usually a crucial prerequisite for its invasive and metastatic potential 17. Therefore, investigation of potential key molecules involved in EMT properties may contribute to develop new therapeutic strategies against HCC invasion and metastasis.

Mammalian sterile-20-like kinase 4 (MST4) is a member of the germinal center kinase (GCK) group III family of kinases. Studies have shown that MST4 affects a variety of physiological functions, including Golgi reorientation, autophagy, intestinal epithelial cell brush border formation, gastric parietal cell polarized epithelial secretion, and inflammatory response 19-22. The functional role of MST4 in cancers has not been fully elucidated and remains controversial. It is reported that MST4 facilitates the growth of prostate cancer, pancreatic cancer, and glioblastoma 23, 24. In breast cancer and gastric cancer, the expression of MST4 is associated with metastasis and poor prognosis 25, 26. Another study showed that MST4 can inhibit gastric carcinogenesis by phosphorylating and inactivating YAP oncoprotein 27. In choriocarcinoma, MST4 is shown to inhibit cell migration and invasion by suppressing the TGF-β1-induced EMT program 28. In our recent work, we found that MST4 is down-regulated in HCC, and that it suppresses HCC cell proliferation and cell cycle progression by inactivating PI3K/AKT pathway 29. However, as the major cause of HCC progression and poor prognosis, the role of MST4 in the regulation of invasion and metastasis of HCC is worth further exploration.

In this study, we found that the expression of MST4 is negatively correlated with the progression and poor prognosis of HCC. Functional inactivation of MST4 can promote the migration, invasion, and metastasis of HCC cells, whereas the overexpression of MST4 has the opposite effect. Mechanistically, we found that MST4 inhibits the motility and invasive potential of HCC cells by inactivating PI3K/AKT signaling pathway, which leads to the decrease of Snail1 expression, thus blocking the EMT phenotype. Collectively, our results revealed that MST4 is an important prognostic biomarker for aggressive invasion and IM in patients with HCC, and provides new insights into the role and molecular mechanism of MST4 in HCC progression.

## Materials and methods

### Clinical specimens

A total of 335 formalin-fixed paraffin-embedded HCC biopsies were obtained from the Department of Pathology, the Second Affiliated Hospital of Guilin Medical College, China. 85 HCC patients who had complete follow-up data were enrolled for survival analysis. All HCC patients enrolled in this study were histopathologically and clinically diagnosed. None of these patients received preoperative radiotherapy or chemotherapy. The clinical information of 10 SHC patients is presented in Table S1. Retrieval of tissue and clinical data was performed according to the regulations of the institutional review boards (IRB) of the Second Affiliated Hospital of Guilin Medical University and data safety laws with specific regard to ethical standards and patient confidentiality. The patients whose tissues were used provided written informed consent. Ethical approval was given by the Medical Ethics Committee of Southern Medical University. The study is compliant with all relevant ethical regulations involving human participants.

### Histological analysis and immunohistochemistry (IHC)

Histological analysis and immunohistochemical staining were performed as described previously 30. Formalin-fixed, paraffin-embedded tumor tissues from patients with HCC were collected. The paraffin sections were deparaffinized in xylene and rehydrated in ethanol with decreasing concentrations. The antigen retrieval was performed in a steam pressure cooker with citrate buffered saline (pH 6.0) for 3 minutes. Endogenous peroxidase was blocked by incubation with 3% hydrogen peroxide for 30 minutes. After incubation with 5% normal goat serum for 30 minutes at room temperature to block unspecific labeling, sections were incubated overnight in humidified chamber at 4 °C with primary antibodies (Table S2). The slides were then stained with a corresponding secondary antibody at 37 °C for 20 minutes. Finally, the sections were stained with diaminobenzidine (DAB) and counterstained with hematoxylin. Histopathology and immunohistochemistry analyses were performed independently by two pathologists without knowledge of the backgrounds of the patients. Staining intensity and percentage of staining-positive cells were evaluated for the IHC scores. Staining intensity was classified as 0 (negative), 1 (weak), 2 (moderate) and 3 (strong). Percentage of stained cells were classified as 0 (<5 %), 1 (≥ 5%, <25 %), 2 (≥25, <75%) and 3 (≥75%). The final IHC score is score of intensity multiplied by score of percentage. The scores 0, 1, 2, 3, 4 were defined as low, and the scores 6 and 9 were defined as high. Slides with conflicting evaluations were reassessed, and a consensus was reached.

### Cell lines and cell culture

HCC cell lines (Bel-7402, Bel-7404 and Huh7) and HEK-293T cells were purchased from the Cell Bank of Shanghai Institutes for Biological Sciences (SIBS), Chinese Academy of Sciences (CAS) (Shanghai, China) or ATCC. All cells were cultured in Dulbecco's modified Eagle medium (DMEM) (10-013-CVR, Corning) supplemented with 10% fetal bovine serum (ST30-3302, PAN) at 37 °C under 5% CO_2_ in a humidified chamber.

### Plasmid constructs and interfering RNAs

The pcDNA3.1-MST4-WT and pcDNA3.1-MST4-T178A (dnMST4: a dominant negative mutant of MST4-WT) were kindly provided by Prof. Hans Clevers 19. The lentiviral pLV-MST4 and pLV-dnMST4 plasmids were generated by subcloning the MST4-WT fragments (from pcDNA3.1-MST4-WT) and MST4-T178A fragments (from pcDNA3.1-MST4-T178A) into pCDH-EF1-GFP-Puro (System Biosciences, Palo Alto, CA, #CD550A-1) lentiviral vectors. The lentiviral packaging plasmids psPAX2 and pMD2.G were kindly provided by Prof. Didier Trono (University of Geneva, Geneva, Switzerland). For RNA interference in HCC cells, specific siRNAs were used for Snail1 (Snail1 siRNA (antisense): UGGCACUGGUACUUCUUGACAUCUGTT.

### Virus packaging and infection

The pLV-MST4, pLV-dnMST4 or pCDH-EF1-GFP-puro empty vector was respectively co-transfected into HEK-293T cells with lentiviral packaging plasmids (psPAX2 and pMD2.G) to produce the lentiviruses. The viral supernatants were harvested at 72 hours after transfection, and subsequently used to infect the indicated cells to generate vector-, MST4- or dnMST4-expressing cancer cell lines, respectively.

### Western blot assay

Western blotting was performed as previously described 29. Briefly, the same amount of total denatured protein (20 μg) was isolated by 10% SDS-PAGE and transferred to PVDF membranes (P2120-2, Millipore) using BIO-RAD system. After blocking for nonspecific binding using 5% skim milk, the membranes were then incubated with primary antibodies (Table S3) overnight at 4 °C, followed by incubation with an HRP-labeled goat anti-rabbit IgG or anti-mouse IgG for one hour at room temperature. After washing four times in TBST, protein bands were visualized on a chemiluminescence imaging system (ChemiDoc XRS+, Bio-Rad).

### Transwell migration and invasion assay

Approximately 1×10^5^ HCC cells were suspended in serum-free medium and added in either top-chambers that were not coated with Matrigel for migration assay or Matrigel-coated chambers with 8 μm pores (3422, Corning Costar) for invasion assay. The culture medium containing 10% FBS was added to the bottom chamber. After incubation at 37 °C for eighteen hours, HCC cells may migrate to the bottom chamber through the membrane. Membranes were washed three times with 1×PBS, fixed in 4% paraformaldehyde, and stained with crystal violet staining solution (1.09218, Sigma). The number of migrated or invaded cells through the membrane was separately counted under the microscope from 10 random fields of view.

### Sphere formation assay

The HCC cells (1×10^5^) were cultured in sphere medium and plated in 6-well ultra-low cluster plates. The sphere medium was composed of DMEM:F-12 serum-free medium supplemented with 20 ng/ml rhEGF (Peprotech), 20 ng/ml rbFGF (Peprotech), 1% B-27 (17504044, Gibco), and 2 mg/ml heparin (H3149, Sigma) 31.The HCC cells were cultured in sphere medium at 37 °C in 5% CO_2_, and counted floating spheres after ten days. The representative images were taken with Nikon microscopes. Every experiment was carried out multiple independent experiments.

### Immunofluorescence (IF) staining

IF staining was performed according to the protocol of a standard method described previously 32. The slides were counterstained with DAPI (Sigma) for five minutes to visualize the nuclei and imaged with a confocal laser-scanning microscope (Nikon A1). The primary antibodies used for IF staining were listed in Table S2.

### PI3K inhibitor treatment

For long-term inhibitor treatment, 10 μM PI3K inhibitor LY294002 (Catalog # HY-10108; MedChemExpress (MCE) was added in the cultured cells every 3 days due to the half-life of LY294002.

### *In vivo* metastasis analysis in nude mice

BALB/c nude mice (3-4 weeks) were purchased from the Animal Center of Southern Medical University and raised in a pathogen-free environment. For *in vivo* metastasis assays, the vector- or dnMST4-expressing Bel-7402 cells (1.0×10^6^) were subcapsularly transplanted into the liver of nude mice, respectively. All animals were sacrificed after transplantation, and liver were collected for metastasis analysis. This study was carried out in accordance with the Guide for the Care and Use of Laboratory Animals of the Southern Medical University. The protocol was approved by the Committee on the Ethics of Animal Experiments of the Southern Medical University. Subcapsular cell transplantation was performed under pentobarbital sodium anesthesia to minimize mice suffering.

### Statistical analysis

The data were presented as mean ±SEM from three independent experiments. Statistical analyses were conducted using the SPSS 20.0 software package and Graphpad 8.0 software. A two-tailed Student's t test was used for comparisons of two independent groups. The χ^2^ test was used to analyze the association between clinicopathological characteristics and MST4 expression. Overall survival (OS) was measured from the onset of treatment to the date of death or the survival status at the last date of follow-up. OS probabilities were estimated by the Kaplan-Meier method and the significance of differences was assessed by the log-rank test. Values are statistically significant at *P<0.05, **P<0.01 and ***P<0.001.

## Results

### Low MST4 expression correlates with HCC progression and poor prognosis

**Figure 1:**
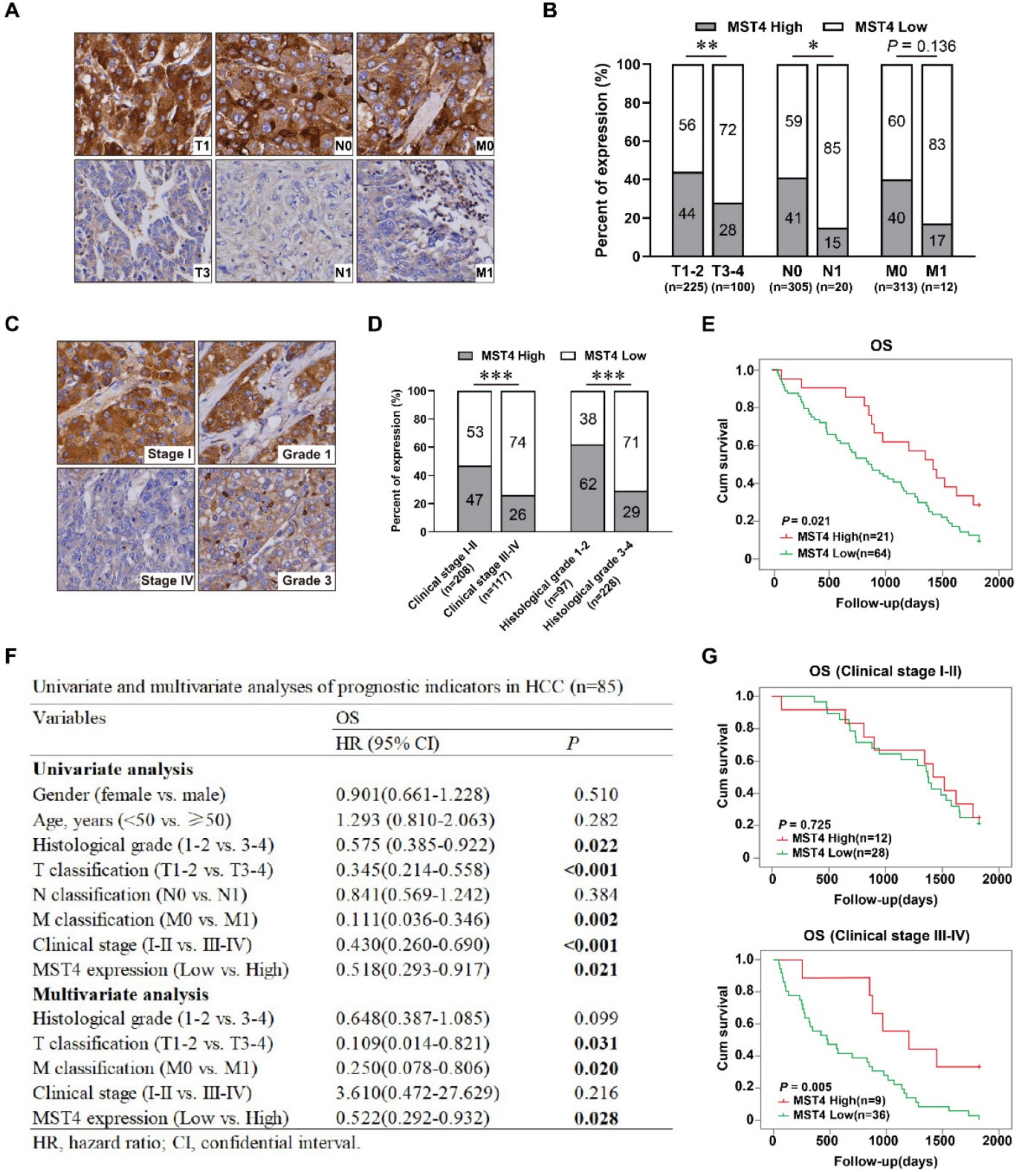
** Low MST4 expression correlates with HCC progression and poor prognosis. (A)** Representative images of MST4 immunohistochemical (IHC) staining in HCC specimens (n=325) with different TNM stages. **(B)** The percentages of cases with high or low expression of MST4 according to different TNM stages of HCC. **(C)** Representative images of MST4 IHC staining in HCC specimens (n=325) with different clinical stages and histological grades. **(D)** The percentages of cases with high or low expression of MST4 according to different clinical stages and histological grades of HCC. **(E)** Kaplan-Meier survival curves of overall survival (OS) in 85 HCC patients with high MST4 expression (red line) and low MST4 expression (green line) (Log-rank test; *P*=0.021). **(F)** Univariate and multivariate analyses of prognostic indicators in HCC (n=85). **(G)** The correlation of MST4 expression with survival time for HCC patients stratified by clinical stage I-II (upper panel) (Log-rank test; *P*=0.725) and clinical stage III-IV (lower panel) (Log-rank test; *P*=0.005). Data are mean ± SEM (*P< 0.05, **P <0.01, ***P <0.001).

Our recent work showed the expression of MST4 in HCC is lower than that in paracancerous liver tissues, and MST4 has an anti-tumorigenic effect on HCC 29. For patients, HCC with high invasiveness and metastasis usually leads to rapid progression and indicates a poor prognosis. Higher AJCC T-stage based on larger tumor size, microvascular invasion, portal or hepatic vein invasion, and adjacent organ invasion. N-stage and M-stage define regional lymph nodes and distant metastasis, respectively. To determine the clinical relevance of MST4 in HCC progression, we examined the expression of MST4 in 325 paraffin-embedded human HCC specimens by immunohistochemical analysis. Our data showed the frequency of MST4 expression was significantly higher in T1-2 stage (99/225, 44%) than in T3-4 stage (28/100, 28%), and in N0 stage (124/305, 41%) than in N1 stage (3/20, 15%) of HCC. A similar trend was found in comparison MST4 expression in M0 and M1 stages (despite this not being statistically different) (Figure 1A, 1B and Table 1).

The clinical stage according to the AJCC TNM staging system is of great significance for predicting the prognosis of HCC patients and determine the optimal clinical treatment. The histological grade is assigned by the degrees of HCC differentiation (according to the Edmondson Steiner grading system), and serves as an independent prognostic indicator for HCC patients. Our study demonstrated a significantly higher frequency of MST4 expression in HCC patients with low clinical stage (stage I-II; 97/208, 47%) than those with high clinical stage (stage III-IV; 30/117, 26%), and in patients with well or moderately differentiated HCC (histological grade 1-2; 60/97, 62%) than those with poorly differentiated HCC (histological grade 3-4; 67/228; 29%) (Figure 1C, 1D and Table 1), implicating MST4 may serve as a prognostic indicator for HCC.

Further we studied the prognostic value of MST4 based on clinical follow-up data. Kaplan-Meier survival analysis showed that HCC patients with high MST4 expression has a notably longer overall survival (OS) than those with low MST4 expression (*P*=0.021) (Figure 1E). Univariate and multivariate analyses revealed that MST4 expression is an independent prognostic factor for OS (*P* = 0.028, HR = 0.522) in HCC patients (Figure 1F). Interestingly, for patients with stage III-IV, the OS of the MST4 high group was significantly longer than that of the MST4 low group (*P*=0.005) (Figure 1G), whereas for patients with stage I-II, the differences in OS between the high group and the low group were not statistically significant (Figure 1G). Overall, these results suggested that low MST4 expression is closely correlated with the progression and poor prognosis of HCC, and can be used as a new prognostic biomarker for patients with advanced HCC (stage III-IV).

### MST4 inactivation promotes the migration, invasion, and metastasis of HCC cells

**Figure 2:**
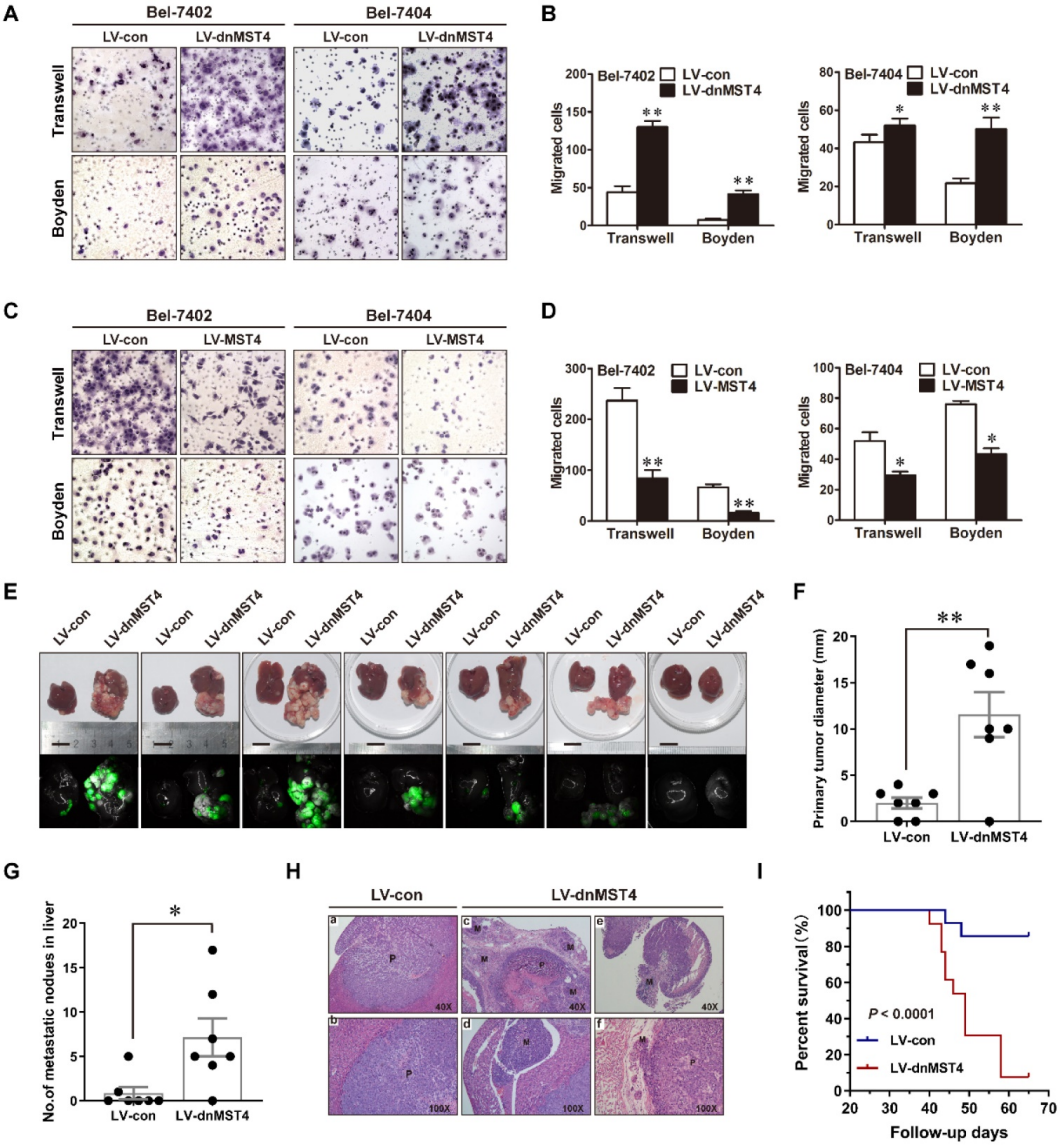
** MST4 negatively regulates HCC cell migration, invasion, and intrahepatic metastasis (IM) *in vitro* and *in vivo*. (A)** Representative photographs of the migratory and invasive properties of dnMST4-expressing Bel-7402 and Bel-7404 cells. **(B)** Quantification of migratory and invasive properties of dnMST4-expressing Bel-7402 and Bel-7404 cells. **(C)** Representative images of the migratory and invasive properties of MST4-overexpressing Bel-7402 and Bel-7404 cells. **(D)** Quantification of migratory and invasive properties of MST4-overexpressing HCC cells. **(E)** Representative pictures of the livers from nude mice after subcapsular liver transplantation of vector-expressing (LV-con) or dnMST4-expressing (LV-dnMST4) Bel-7402 cells (7 mice/group). Photographs were recorded under natural light (upper panel) or under fluorescent stereomicroscope (lower panel). **(F)** The diameter of the primary tumors in the livers of nude mice. **(G)** The number of IM nodules in the livers of nude mice. **(H)** Representative H&E staining of liver tissue samples from nude mice after subcapsular liver transplantation of vector- or dnMST4-expressing Bel-7402 cells. (a and b) primary tumor, (c) satellite nodules near the primary tumor lesion, (d) vascular invasion, (e) lymph node metastasis, and (f) peripheral nerve invasion. P: primary tumor; M: metastatic nodule. **(I)** Survival curve of nude mice transplanted with vector- (bule line) or dnMST4-expressing (red line) Bel-7402 cells into hepatic subcapsular space. Data are mean ± SEM (*P< 0.05, **P <0.01, ***P <0.001).

To determine the role of MST4 in the invasion and metastasis of HCC cells, we performed Transwell migration assays using the dominant-negative mutant MST4 (dnMST4) and the wild-type MST4 stably transfected HCC cells. Our data showed that dnMST4 expression significantly increased the cellular motility of HCC cells (Figure 2A and 2B), whereas forced MST4 expression lowered this ability (Figure 2C and 2D). The invasive potential of tumor cells is a prerequisite for metastasis. Once tumor cells penetrate the basement membrane or extracellular matrix, they journey to new locations directly or through the circulatory system 17. The Boyden assays showed that dnMST4 expression significantly enhanced the invasive potential of HCC cells (Figure 2A and 2B), whereas forced MST4 expression reduced this potential (Figure 2C and 2D).

To further explore the effect of MST4 on the HCC cell metastatic potential *in vivo*, we established orthotopically metastatic models of human HCC in nude mice implanted of dnMST4 and control Bel-7402 cells, respectively (Figure 2E). The orthotopically implanted tumor in the dnMST4 group showed a remarkable larger volume compared to that of the control group (Figure 2F), demonstrating that functional inactivation of MST4 effectively promoted tumor growth. As expected, the number of metastatic nodules in the liver were dramatically increased in the dnMST4 group compared with the control group after *in situ* growth for 6 weeks (Figure 2G). Additionally, hematoxylin and eosin (HE) staining indicated satellite intrahepatic metastatic nodules, tumor thrombus, lymph node involvement, and perineural invasion are detected in the dnMST4 group (Figure 2H), indicating that MST4 functionally loss strengthened the intrahepatic metastatic potential of HCC cells. Given that metastasis is the main cause of death in cancer patients, we asked whether MST4 functionally loss could have an effect on overall survival (OS). The follow-up data revealed that the OS of mice bearing dnMST4 Bel-7402 cells (n=13) was significantly shorter than those bearing control cells (n=14) (Figure 2I), further suggesting the prognostic value of MST4 for HCC. Collectively, these observations indicate that MST4 inactivation promotes the migration, invasion, and metastasis of HCC cells, whereas MST4 overexpression exhibits the opposite phenotypes.

### MST4 repressed the EMT phenotype of HCC cells

**Figure 3:**
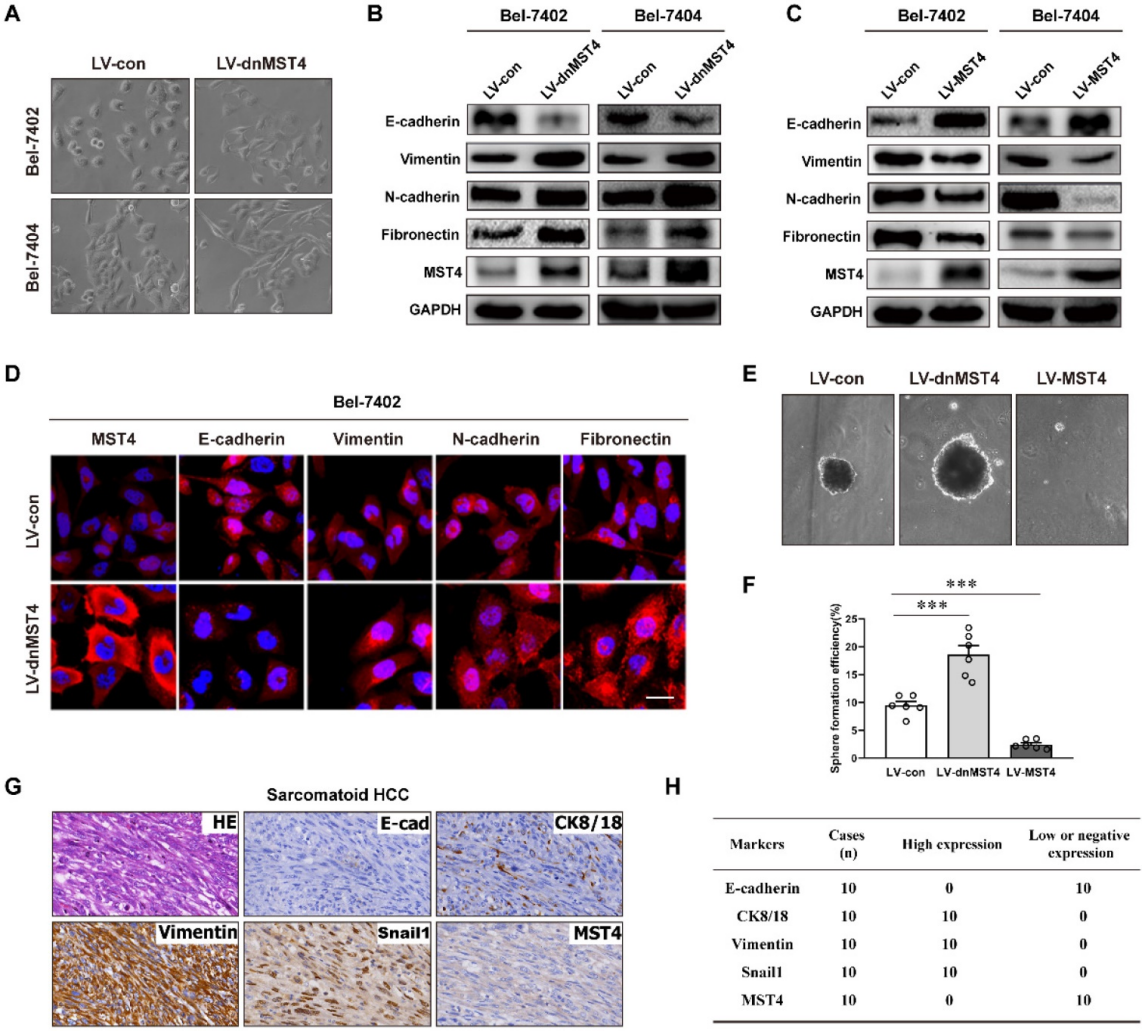
** MST4 represses EMT in HCC cells. (A)** Phase-contrast micrograph of the morphology of vector- or dnMST4-expressing Bel-7402 cells and Bel-7404 cells. **(B)** Western blot analysis for the detection of the indicated protein expression in dnMST4-expressing Bel-7402 cells and Bel-7404 cells. **(C)** Western blot analysis for the detection of the indicated protein expression in MST4-overexpressing Bel-7402 cells and Bel-7404 cells. **(D)** Immunofluorescence images showing the expression of EMT marker proteins of dnMST4-expressing Bel-7402 cells. Blue: DAPI. Scale bar, 20 µm. **(E)** Representative pictures of tumorsphere in dnMST4-expressing and MST4-overexpressing Huh7 cells. **(F)** The number of tumorspheres in dnMST4-expressing and MST4-overexpressing Huh7 cells. **(G)** Representative pictures of H&E and IHC staining for EMT markers in SHC specimens. **(H)** The number of cases with high or low indicated protein expression in SHC specimens. Data are mean ± SEM (*P< 0.05, **P <0.01, ***P <0.001).

It is widely embraced the existence of EMT is essential to enhance tumor progression and metastasis 15, 17, 33-36. Intriguingly, we noticed that the expression of dnMST4 causes significant morphological changes from cobblestone-like to the spindle-shaped appearance in both Bel-7402 and Bel-7404 cells (Figure 3A), indicating that MST4 functionally loss may facilitate the EMT process of HCC cells. EMT markers were then probed by western blot, including epithelial protein E-cadherin and mesenchymal markers N-cadherin, vimentin, and fibronectin. The dnMST4-expression HCC cells showed a typical EMT phenotype, including downregulation of E-cadherin and upregulation of N-cadherin, vimentin, and fibronectin (Figure 3B). Correspondingly, the MST4 overexpression leads to a reversed EMT phenotype, including the increase of E‑cadherin and the decreases of N-cadherin, vimentin, and fibronectin (Figure 3C). The phenotype was further confirmed by immunofluorescent staining (Figure 3D). It is known that cancer cells undergo EMT can also acquire stemness properties 37, so we investigated whether the MST4 functionally inactivation promotes the stemness of HCC cells. As expected, the expression of dnMST4 significantly increased the sphere-forming efficiency (SFE), which can be used to evaluate the population of cancer stem-like cells (CSLCs) 38, whereas the forced expression of MST4 reduced that (Figure 3E and 3F). Sarcomatoid hepatocellular carcinoma (SHC) is a rare type of HCC with positive expression of vimentin and predominantly composed of anaplastic spindle-like cells 39-41. CK8 positive staining is used for differentiating SHC from real sarcomas 40. EMT is implicated in the development of this cancer 42. Immunohistochemical analysis showed that the expression of MST4 was negative or low in all 10 cases of SHC (Figure 3G and 3 H), which indicated a negative correlation between the EMT phenotype and MST4 expression in HCC cells. Collectively, our results supported the inhibitory potential of MST4 expression against the EMT phenotype of HCC cells.

### MST4 inactivation induces the EMT phenotype of HCC cells, and promotes their invasive potential through activation of PI3K/AKT/Snail1 signaling pathway

**Figure 4:**
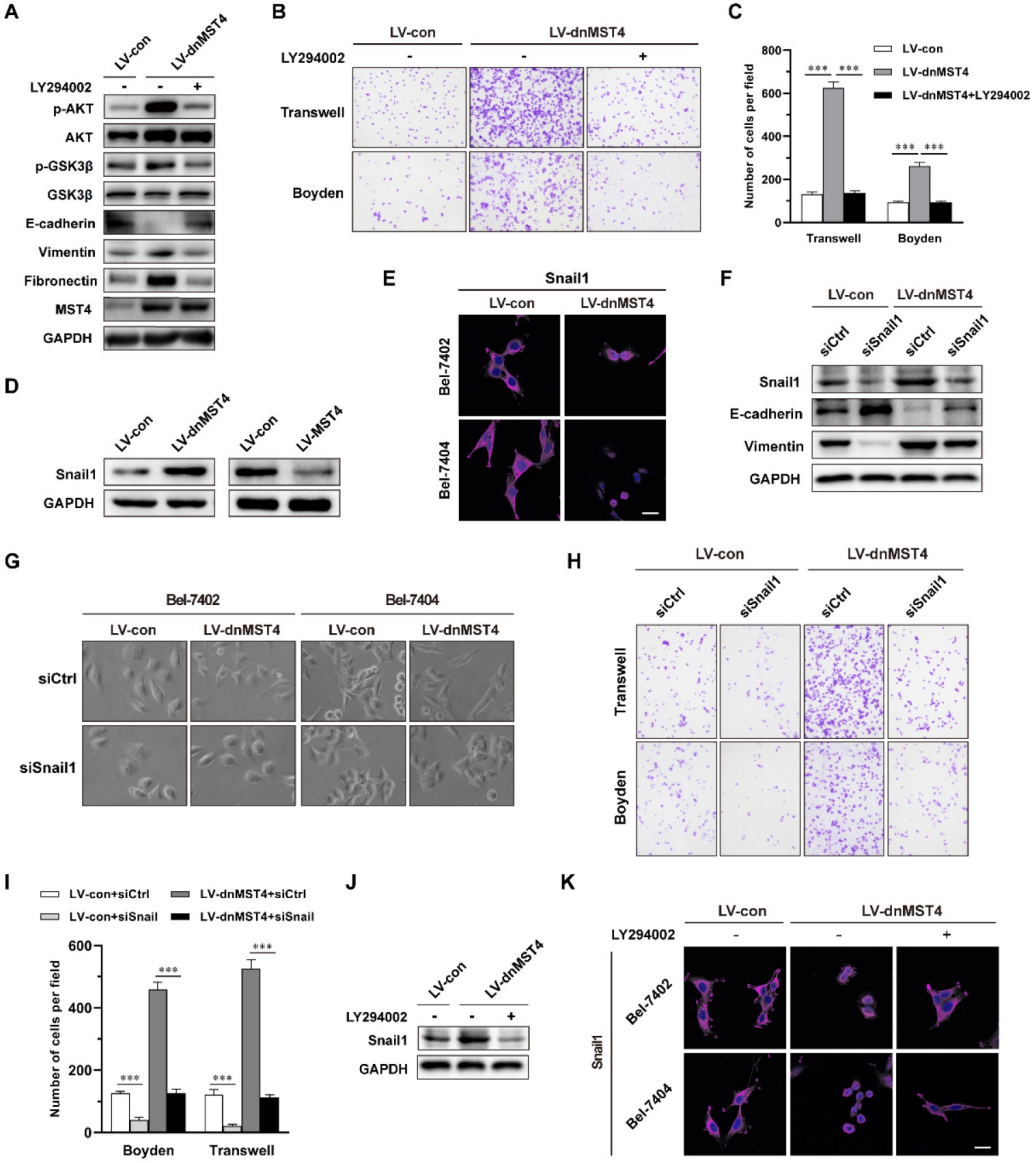
** MST4 suppresses EMT and invasion of HCC cells through inactivation of PI3K/AKT/Snail1 signaling axis. (A)** Western blot analysis for indicated protein levels in dnMST4-expressing Bel-7402 cells treated with or without LY294002 for 24 hours. **(B)** Representative images of migratory and invasive properties of dnMST4-expressing Bel-7402 cells treated with or without LY294002 for 24 hours. **(C)** Quantification of migratory and invasive properties of dnMST4-expressing Bel-7402 cells treated with or without LY294002 for 24 hours. **(D)** Immunoblot analysis of Snail1 protein levels in dnMST4- and LV-MST4-expressing Bel-7402 cells. **(E)** Representative immunofluorescence images for Snail1 levels in vector- and dnMST4-expressing Bel-7402 cells and Bel-7404 cells. Scale bar, 20 µm. **(F)** Immunoblot analysis of the expression of Snail1, E-cadherin and vimentin in vector- and dnMST4-expressing Bel-7402 cells with or without Snail1 knockdown. **(G)** Phase-contrast micrographs of the cell morphology of vector- and dnMST4-expressing HCC cells with or without Snail1 knockdown. **(H)** Representative pictures of the migration and invasion in vector- and dnMST4-expressing Bel-7402 cells with or without Snail1 knockdown. **(I)** Quantification of the migration and invasion in vector- and dnMST4-expressing Bel-7402 cells with or without Snail1 knockdown. **(J)** Western blot analysis of Snail1 expression in dnMST4-expressing Bel-7402 cells with or without LY294002. **(K)** Representative immunofluorescence images for Snail1 in dnMST4-expressing Bel-7402 cells and Bel-7404 cells with or without LY294002. Scale bar, 20 µm. Data are mean ± SEM (*P< 0.05, **P <0.01, ***P <0.001).

Next, we investigated the mechanism by which MST4 suppressed the EMT phenotype and the invasive and metastatic potential of HCC cells. In our previous study, we have unraveled an inhibitory effect of MST4 on PI3K/AKT pathway 29. Here, we investigated whether the inactivation of PI3K/AKT pathway is essential for the suppressed EMT phenotype by MST4 high expression in HCC cells. To this end, we employed a PI3K/AKT inhibitor LY294002, in dnMST4-expressing cells and found that inhibition of the PI3K/AKT pathway could eliminate the up-regulation of vimentin and fibronectin, and down-regulation of E-cadherin induced by dnMST4 expression (Figure 4A). This finding suggested that PI3K/AKT pathway plays a vital role in the MST4-mediated repression of EMT phenotype of HCC cells. In addition, we examined the migration and invasion capability of Bel-7402 cells, and observed that PI3K/AKT inactivation abolished the cell migration and invasion promoted by dnMST4 expression (Figure 4B and 4C), suggesting the necessary role of PI3K/AKT inactivation in MST4 inhibiting the invasive and metastatic potential of HCC cells.

The transcription factor Snail1 is a critical inducer of EMT and plays an important role in cancer invasion and metastasis 43. The activation of AKT can inhibit GSK3β activation, thus stabilize Snail1 and facilitate Snail1 nuclear translocation, promoting EMT and metastasis 44, 45. As shown in Figure 4D, a significant increase in the level of Snail1 was observed in dnMST4-expressing Bel-7402 cells, whereas the MST4 overexpression led to a remarkably reduced level. We also noticed that the expression of dnMST4 caused the translocation of Snail1 into the nucleus (Figure 4E). Next, we examined whether the inhibition of Snail1 is necessary for MST4 to inhibit EMT phenotype, migratory and invasive potential of HCC cells. After the introduction of siRNAs specific for Snail1 into dnMST4-expressing Bel-7402 cells, we analyzed the expression of epithelial and mesenchymal markers. Our results showed that silencing of Snail1 significantly reversed the EMT phenotype induced by dnMST4 expression in Bel-7402 cells, as evidenced by the enhanced expression of the epithelial marker (E-cadherin), and the reduced expression of the mesenchymal marker (vimentin) (Figure 4F). Furthermore, we observed that siSnail1 abolished the spindle-like, fibroblastic morphology and enforced invasive potential promoted by dnMST4 expression in HCC cells (Figure 4G, 4H and 4I). To further elucidate whether the regulatory effect of MST4 on Snail1 is dependent on PI3K/AKT pathway, we applied LY294002 in dnMST4 cells and detected the expression of Snail1. The dnMST4-enforced expression of Snail1 was abolished (Figure 4J) and the location of Snail1 was reversed into cytoplasm (Figure 4K), demonstrating that the effect of MST4 on Snail1 is dependent on PI3K/AKT pathway. Collectively, these results suggested that MST4 inactivation can induce the EMT phenotype of HCC cells, and promote their invasive potential through activation of PI3K/AKT/Snail1 signaling pathway.

### High MST4 expression correlates with the inactivation of PI3K/AKT/Snail signaling and reduced EMT phenotype in HCC patients

**Figure 5:**
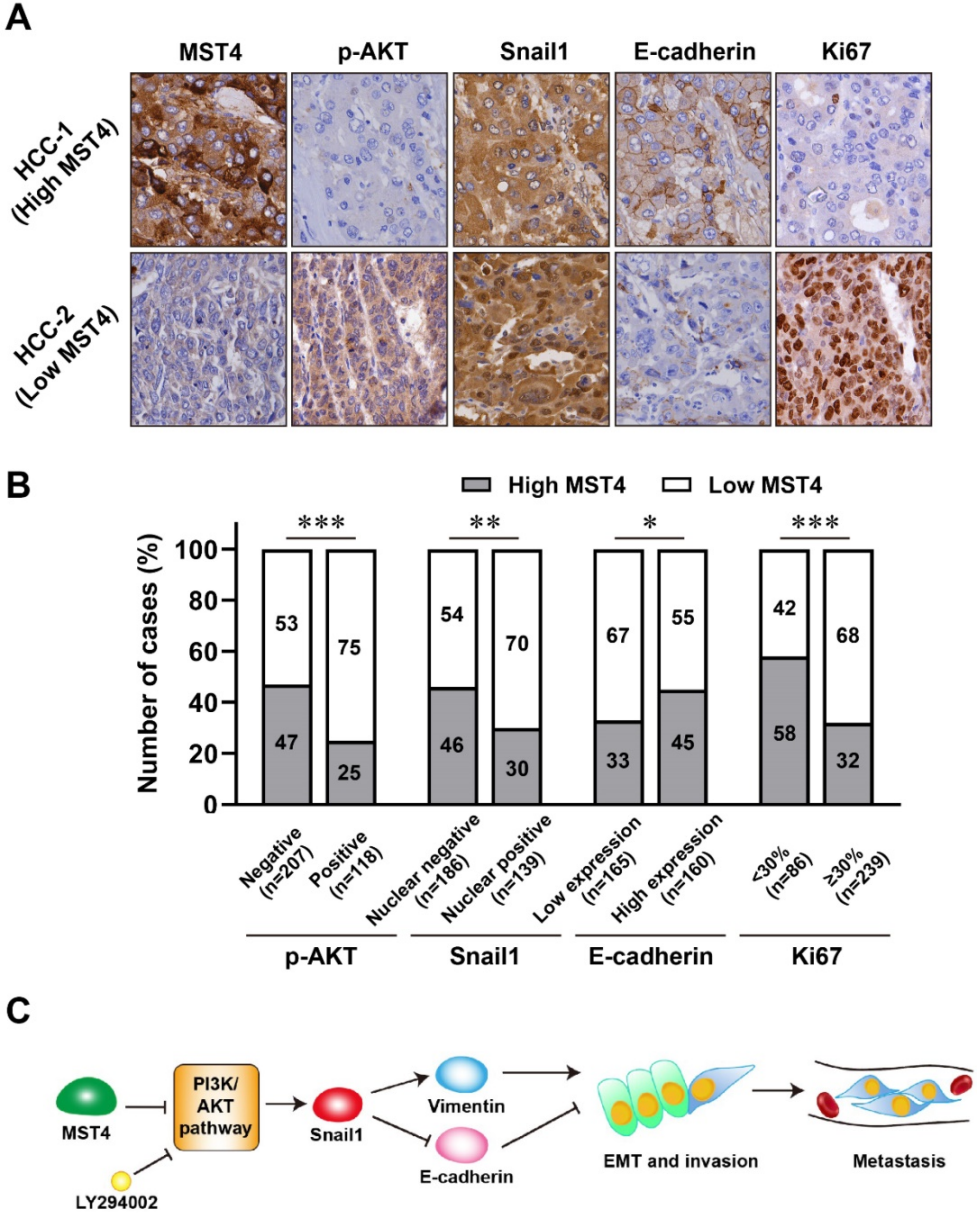
** The expression of MST4, p-AKT, Snail1, E-cadherin and Ki67 in human clinical HCC tissue specimens. (A-B)** Representative images (A) of immunohistochemical staining for MST4, p-AKT, Snail1, E-cadherin and Ki67 in HCC tissues, and statistical analysis across 325 HCC specimens (B). **(C)** A proposed model of MST4 regulating the PI3K/AKT/Snail1 signaling pathway to modulate EMT, invasion and metastasis in HCC cells.

We investigated the expression of MST4, PI3K/AKT/Snail1 pathway components, EMT marker E-cadherin, and proliferation marker Ki67 by immunohistochemical staining in the 325 cases of primary HCC. Representative cases of immunohistochemical staining are shown in Figure 5A. Our results showed that in HCC patients with high MST4 expression, the levels of p-AKT, nuclear Snail1, and Ki67 are down-regulated, while the expression of E-cadherin is up-regulated in these patients (Figure 5A, 5B and Table 2). On the other hand, in patients with low expression of MST4, the levels of p-AKT, nuclear Snail1, and Ki67 were up-regulated, and the typical EMT phenotype characterized by downregulation of E-cadherin were observed.

## Discussion

HCC with a high tendency for invasion and metastasis often indicates poor survival 46. As such, there is an urgent need to identify the key indicators to predict the risk of aggressive invasion and metastasis, with the aim of identifying new therapeutic targets. EMT phenotype is closely related to high invasion, intrahepatic metastasis (IM), and poor prognosis in patients with HCC 47. Here, we disclosed that MST4, a member of the GCKIII kinase family, can suppress the EMT, invasive and metastatic potential of HCC cells by inactivating PI3K/AKT/Snail1 axis, and can act as a potential biomarker for the HCC progression and prognosis.

In clinics, early HCC can be cured by surgical resection. However, recurrence after radical resection often leads to treatment failure and affects the long-term survival of HCC patients 48. Postoperative recurrent HCC (RHCC) can be caused by IM of the primary tumor, with a high incidence and poor prognosis 49, 50. The highly invasive properties of HCC cells into macro- and microvascular vessels can give rise to IM with high frequency 51. Therefore, it is necessary to identify the HCC patients with high invasive and IM potential to increase the chances of curative interventions. In our study, we found that MST4 was robustly down-regulated in HCC with advanced TNM stage, clinical stage, and histological grade. Kaplan-Meier analysis revealed that low MST4 expression correlated with poor prognosis in HCC patients. Strikingly, further subgroup analysis of the patients' clinical stages showed that there was no correlation between the expression of MST4 and OS in patients with I-II stage, whereas in patients with stage III-IV, the OS of patients with low expression of MST4 was significantly worse than that of patients with high expression. By using an orthotopically metastatic model of human HCC in nude mice, we showed that the functional inactivation of MST4 can promote the IM of HCC cells, demonstrating an inhibitory role of MST4 on HCC progression. These findings suggest that MST4 is a potential prognostic indicator for patients with HCC and can be used to predict the potential for HCC aggressive invasion, metastasis, and poor prognosis.

The occurrence of tumor metastasis requires the infiltrating of tumor cells from primary focus into adjacent tissues, trans-endothelial migration into vessels, and survival in the circulation known as anchorage-independent growth 52, 53. Our previous study has shown that MST4 can limit the unanchored growth of HCC cells 29, implicating its potential inhibitory effect on metastasis. In this study, we found that MST4 suppressed the migration and invasion of HCC cells *in vitro*, which represent the initial steps associated with the metastasis 53. Additionally, we observed that the MST4 functionally inactivation promoted the intrahepatic dissemination, lymph node localization, vasculature transmigration, and perineural infiltration of HCC cells in mouse models, and contributed to a worse prognosis. These observations indicate that MST4 plays an important role in blocking the invasive and metastatic progression of HCC. However, the specific mechanism of each step in this process may be complex and different, which is worthy of our further exploration.

EMT is a process of cell de-differentiation from epithelial into mesenchymal phenotype. In HCC, EMT can reduce the cell contacts and gain increased motility, thus causing cell spreading 46. Multiple studies clarified the correlation between EMT and a high tendency to migrate, invade, and metastasize. An early study of 72 HCC patients with chronic liver disease showed that the E-cadherin expression in metastatic HCC was lower than that in non-metastatic HCC 54. Another immunohistochemical study established the decreased expression of E-cadherin was significantly correlated with IM and capsular invasion in HCC 55, 56. Our clinical data showed that MST4 was inversely correlated with the histological grade of HCC, which was based on the degree of differentiation 55, indicating a potential relevance of MST4 in the EMT of HCC. Further immunohistochemical analysis revealed that MST4 expression was positively correlated with E-cadherin and reversely correlated with E-cadherin transcriptional repressor Snail1. In our 10 cases of SHC, which expressed EMT-related markers Vimentin and Snail1 and lacked E-cadherin expression, loss of MST4 is observed. These results confirmed a significant negative correlation between MST4 expression and EMT status in HCC. In addition, our mechanistic study uncovered that inhibition of MST4 promoted the typical EMT phenotype, while MST4 overexpression reduced this phenotype. Above all, our data demonstrate that MST4 may affect the HCC aggressiveness and metastasis by altering the EMT status.

Another crucial finding of our work is that the inactivation of PI3K/AKT signaling is the cause of MST4-mediated inhibition of EMT, invasion, and metastasis in HCC. The PI3K/AKT signaling plays an important role in regulating cell proliferation and maintaining multiple biological characteristics of malignant cells 57, 58. An early study has established that PI3K/AKT activation is essential for the EMT and cell migration in tumor cells induced by TGF-β1 59. Another finding reported that PI3K/AKT can sustain NF-kB activation in the absence of TGF-β1, thus inducing EMT and promoting tumor formation and metastasis 60. Our previous study illustrated that MST4 inhibits HCC cell proliferation by inactivating the PI3K/AKT signaling pathway 29. Here, our data further confirmed that the PI3K inhibitor can reverse the EMT phenotypic change and the increased capabilities of migration and invasion of HCC cells caused by the MST4 functionally inactivation. Therefore, we can conclude that the inhibition of PI3K/AKT signaling pathway is also essential for MST4 to inhibit the EMT phenotype and capabilities of invasion and metastasis of HCC cells.

Snail1 is a zinc-finger protein that binds to E-box, an E-cadherin promoter region, and represses the expression of the adhesion molecule, thereby breaking the cell-cell tightly contacts and triggering the EMT phenotypic change 45, 61. The nuclear localization of Snail1 facilitates the expression of mesenchymal markers, such as vimentin and fibronectin, completes the EMT and increases migration, invasion, and metastasis of tumor cells 62-64. PI3K/AKT signaling pathway can regulate Snail1 intracellular expression through multiple mechanisms 45. For instance, PI3K/AKT can phosphorylate GSK-3β at serine of the 9th residue, leading to its ubiquitination and degradation, thereby blocking the phosphorylation of Snail1 mediated by GSK-3β and increasing its stability and location in the nucleus 44. From our results, the phosphorylation levels of AKT and GSK-3β were upregulated by MST4 inactivation, indicating Snail1 may function as a downstream target of MST4 in HCC. By using siRNA against Snail1, we confirmed that the reduced expression of Snail1 is responsible for the attenuated EMT, migration and invasion of HCC cells triggered by MST4. Furthermore, using the PI3K inhibitor, we proved that the PI3K/AKT signaling pathway is the hinge between MST4 and Snail1. Therefore, we suggest that through inactivation of PI3K/AKT signaling pathway, MST4 suppressed Snail1 expression, which led to the upregulation of epithelial marker E-cadherin and downregulation of mesenchymal markers vimentin and fibronectin, thus inhibiting the potential of EMT, invasion, and metastasis of HCC cells (Figure 5C).

Based on our mechanistic studies, we further detected the relative expression levels of MST4, PI3K/AKT/Snail1 pathway components, as well as the EMT marker E-cadherin in HCC patients. We found that the frequency of PI3K/AKT/Snail1 pathway inactivation and elevated level of the epithelial marker E-cadherin was higher in HCC patients with high MST4 expression. In contrast, patients with low MST4 expression had a higher frequency of PI3K/AKT/Snail1 axis activation and EMT progression characterized by E-cadherin down-regulation. Taken together, our findings suggest that MST4 serves as a tumor suppressor on HCC cell aggressive invasion and metastasis, and its low expression is a potential biomarker for the HCC poor prognosis.

## Supplementary Material

Supplementary tables.

## Figures and Tables

**Table 1:** Correlation between the clinicopathological features and MST4 expression in 325 HCC tissue specimens

Variables	Case No.	MST4 expression		χ^2^	*P*
	(n)	High (n, %)	Low (n, %)		
**Gender**					
Female	60	20 (33.3)	40 (66.7)	0.745	0.38
Male	265	107 (40.4)	158 (59.6)		
**Age (years)**					
<50	158	57 (36.1)	101 (63.9)	0.931	0.307
≥50	167	70 (41.9)	97 (58.1)		
**Histological grade**					
I-II	97	60 (61.9)	37 (38.1)	28.79	**<0.001**
III-IV	228	67 (29.4)	161 (70.6)		
**T classification**					
T1-T2	225	99 (44.0)	126 (56.0)	6.788	**0.007**
T3-T4	100	28 (28.0)	72 (72.0)		
**N classification**					
N0	305	124 (40.7)	181 (59.3)	5.189	**0.031**
N1	20	3 (15.0)	17 (85.0)		
**M classification**					
M0	313	125 (39.9)	188 (60.1)	2.629	0.136
M1	12	2 (16.7)	10 (83.3)		
**Clinical stage**					
I-II	208	97 (46.6)	111 (53.4)	13	**<0.001**
III-IV	117	30 (25.6)	87 (74.4)		

T: tumor size; N: lymph node metastasis; M: distant metastasis.

**Table 2:** Correlation between MST4 expression and p-AkT level, Snail1 expression, E-cadherin expression & Ki67 in 325 HCC tissue specimens

Markers	Case No.	MST4 expression		χ^2^	*P*
	(n)	High (n, %)	Low (n, %)		
**p-AkT**					
Negative	207	98 (47.34)	109 (52.66)	15.421	**<0.001**
Positive	118	29 (24.58)	89 (75.42)		
**Snail1**					
Nuclear negative	186	85 (45.70)	101 (54.30)	7.373	**0.003**
Nuclear positive	139	42 (30.22)	97 (69.78)		
**E-cadherin**					
Low	165	55 (33.33)	110 (66.67)	4.167	**0.041**
High	160	72 (45.00)	88 (55.00)		
**Ki67 index (%)**					
<30%	86	50 (58.14)	36 (41.86)	16.778	**<0.001**
≥30%	239	77 (32.22)	162 (67.78)		
