# Supplementary material for: MST4 negatively regulates the EMT, invasion and metastasis of HCC cells by inactivating PI3K/AKT/Snail1 axis
Source: J Cancer. 2021 May 27;12(15):4463–77. doi: 10.7150/jca.60008 (PMC8210547; doi:10.7150/jca.60008)
Supplement: Supplementary file 1 — Supplementary tables. [file jcav12p4463s1.pdf]

Supplementary Information

Table S1 Overall survival in patients with sarcomatoid HCC

Case No.	Sex	Age (years)	Overall survival (months)
1	M	46	3
2	M	69	12
3	F	37	1.5
4	M	59	2.5
5	M	71	5
6	M	48	14
7	F	53	16
8	M	65	2
9	M	42	4
10	M	61	8
Ratios or median	F:M = 2:8	56 (37-71)	4.5 (1.5-16)

Table S2 List of antibodies and suppliers

used for immunohistochemistry (IHC) or immunofluorescence (IF)

Antibody	Isotype	Suppliers	Cat. No	Application
MST4	Rabbit IgG	Abcam	ab52491	WB、IHC、IF
Ki67	Rabbit IgG	Thermo Fisher SCIENTIFIC	PA5-19462	IHC
CK8/18(CAM5.2)	Mouse IgG	BD Biosciences	349205	IHC
E-cadherin	Mouse IgG	Santa Cruz	sc-21791	IHC
Vimentin	Mouse IgG	Santa Cruz	sc-6260	IHC
Snail1	Goat IgG	Abcam	ab53519	WB、IHC、IF
E-cadherin	Mouse IgG	BD Biosciences	610182	WB、IF
N-cadherin	Mouse IgG	BD Biosciences	610920	WB、IF
Vimentin	Mouse IgG	BD Biosciences	562337	WB、IF
Fibronectin	Mouse IgG	BD Biosciences	610077	WB、IF
p-AKT(S473)	Rabbit IgG	CST	4060S	WB、IHC

Table S3 List of antibodies and suppliers used for immunoblotting

Antibody	Isotype	Suppliers	Cat. No	Application
MST4	Rabbit IgG	Abcam	ab52491	WB、IHC、IF
Snail1	Goat IgG	Abcam	ab53519	WB、IHC、IF
Snail1	Rabbit	Cell Signaling Technology (CST)	3879S	WB
E-cadherin	Mouse IgG	BD Biosciences	610182	WB、IF
N-cadherin	Mouse IgG	BD Biosciences	610920	WB、IF
Vimentin	Mouse IgG	BD Biosciences	562337	WB、IF
Fibronectin	Mouse IgG	BD Biosciences	610077	WB、IF
E-cadherin	Rabbit IgG	Proteintech	20874-1-AP	WB、IF
N-cadherin	Rabbit IgG	Proteintech	22018-1-AP	WB、IF
Vimentin	Rabbit IgG	Proteintech	10366-1-AP	WB、IF
Fibronectin	Rabbit IgG	Proteintech	15613-1-AP	WB、IF
p-Akt (S473)	Rabbit IgG	CST	4060S	WB、IHC
Akt (pan)	Rabbit IgG	CST	4685S	WB
p-GSK-3-beta(S9)	Rabbit IgG	CST	9323S	WB
GSK-3-beta	Rabbit IgG	CST	9315S	WB
GAPGH	Rabbit IgG	Proteintech	10494-1-AP	WB
